# Videolaryngoscopy as a primary intubation modality in obstetrics: A narrative review of current evidence

**DOI:** 10.17305/bb.2023.9154

**Published:** 2023-12-01

**Authors:** Tatjana Stopar Pintarič

**Affiliations:** 1 Department of Anaesthesiology and Intensive Therapy, University Medical Centre Ljubljana, Ljubljana, Slovenia; 2 Institute of Anatomy, Medical Faculty, University of Ljubljana, Ljubljana, Slovenia

**Keywords:** Videolaryngoscopy, direct laryngoscopy, endotracheal intubation, obstetric anaesthesia, airway management

## Abstract

Pregnancy-related physiologic and anatomic changes affect oxygenation and airway management, and it is widely believed that airway difficulty may be more common in obstetric patients as a result. In addition, most obstetric intubations are performed under emergency conditions, and preoperative airway assessment poorly predicts airway management outcomes. These considerations necessitate special protocols for airway care in the obstetric population, and the evolution of the videolaryngoscope represents one of the most important milestones in recent decades. However, recommendations for the use of videolaryngoscopy in obstetrics remain unclear. A considerable body of evidence affirms that videolaryngoscopy improves laryngeal visualization, increases first-attempt and overall intubation success rates, shortens intubation time, and facilitates team communication and education. In contrast, a significant number of studies have also reported conflicting results regarding comparative clinical outcomes and have highlighted other limitations regarding the adoption of videolaryngoscopy in routine obstetric care. Nevertheless, considering the peculiarities of obstetric intubation, the Macintosh-style videolaryngoscope can be suggested as the primary intubation device as it offers the benefits of both videolaryngoscopy and direct laryngoscopy. However, more rigorous evidence is needed to clarify the current blind spots and controversies regarding the role of videolaryngoscopy in obstetrics.

## Introduction

The obstetric airway presents unique anatomic and physiologic challenges that affect anesthetic outcomes, and despite recent advances in difficult airway management, failed intubation/ventilation remains an important cause of general anaesthesia-related morbidity and mortality in pregnant women [1, 2]. In the United States, the case fatality rate for general anesthesia administered for cesarean delivery was estimated at 6.5 per million anesthetics [3], while in low- and middle-income countries, approximately one in seven maternal deaths during or following cesarean section were attributed to anesthesia, mostly general anesthesia [4]. A UK national survey of the incidence of failed tracheal intubation during obstetric general anesthesia reported a rate of 1 in 224 [5]. In 2015, Kinsella et al. [6] reported that the incidence of failed tracheal intubation has remained stable over the past 4 decades at 2.6 (1 in 390) and 2.3 (1 in 443) per 1000 anesthetics for obstetric general anesthesia and cesarean section, respectively. In a recent multicentre study involving approximately 14,000 general anesthetics for cesarean delivery, the overall risks of difficult and failed intubation were reported as 1:49 and 1:808, respectively [7]. In comparison, the incidence of difficult and failed intubation in the general surgical population was estimated at 1:385 and 1:2230 patients, respectively [8, 9]. Maternal death from failed intubation in cesarean delivery was estimated at 1 per 90 failed intubations or 2.3 per 100,000 general anesthetics, with aspiration and hypoxemia being the leading cause of mortality [6]. Failed intubation was also an independent predictor of neonatal intensive care unit admission [6].

## Peculiarities of the obstetric airway

The physiologic and anatomic changes related to pregnancy affect oxygenation and airway management [2], and the presence of co-existing morbidities, such as obesity or preeclampsia, may further complicate anesthetic care. Upper airway edema and friability in pregnancy decrease the pharyngolaryngeal tract diameter, reduce airway compliance and increase the risk of mucosal bleeding and difficulty in passing the endotracheal tube [10–13]. Fluid retention and weight gain correlate with an increase in the Mallampati score [14]. Reduction in functional residual capacity combined with a rise in oxygen consumption at term and in labor lowers the oxygen reserve, accelerating the occurrence of oxygen desaturation during hypoventilation and apnoea [10, 15]. In addition, pregnancy is associated with decreased gastric emptying, gastric pH, and gastro-esophageal sphincter competence and increased intragastric pressure and risk of inhalation of gastric contents [10, 13].

On the other hand, the incidence of airway management complications is likely to escalate with the declining trend of experience and skills in obstetric general anesthesia. The bulk of “first on” obstetric anesthetic cover is often provided by specialist registrars, senior house officers, and non-trainees with limited exposure and experience in obstetric general anesthesia, mostly in the context of obstetric emergencies and often outside of regular working hours [16–18]. Besides, obstetric operating rooms are often remotely located and may not always have the resources necessary to handle a difficult airway [19]. In smaller, understaffed obstetric units, there may be a higher likelihood of inadequate clinical or equipment preparedness and assessment under emergency circumstances.

Several studies have attempted to characterize the risk factors for failed or difficult intubation in the parturient. Reale et al. [7] found that non-obstetric factors, such as increased body mass index (BMI), Mallampati score III or IV, short hyoid-to-mentum distance, and limited jaw protrusion, mouth opening, and cervical spine accounted for most of the risk factors for intubation difficulties in parturients. A UK survey similarly reported that failed tracheal intubation was independently predicted by age, BMI, and a documented Mallampati score [5]. The incidence of failed intubations was found to be disproportionately higher in Asians and Africans/Afro-Caribbeans, reflecting the increased use of general anesthesia in these patients [16], suggesting the importance of racial demographics as a predictive factor. However, in practice, failed or difficult intubation in the parturient is often unpredictable [10] because clinical screening tests, such as neck circumference, sternomental distance, the modified Mallampati test, and the ratio of neck circumference to sternomental distance, are of limited predictive value [20–22].

In contrast to common belief [8, 23], several recent studies found no differences in general anesthetic outcomes between obstetric and non-obstetric patients. For example, a retrospective analysis of 2802 obstetric patients from 2010 to 2015 found that regardless of the increase in obesity and the American Society of Anaesthesiologists Physical Status (ASA) scores, the incidence of failed intubation was not increased in obstetric patients [24]. Similarly, the incidence of failed intubations was found to be similar in obstetric patients undergoing surgical delivery and non-pregnant female patients undergoing non-obstetric abdominal or gynecologic surgery under general anesthesia [25]. Besides methodological differences and limitations, the discrepancies in the literature regarding the incidence of difficult or failed intubation in obstetric versus general population may reflect the improved understanding and management practices for the difficult airway, enhanced clinical screening, and increased use of neuraxial techniques in women with predicted airway difficulty, or institution-specific factors, such as availability of experienced teams and modern equipment, and adherence to standard guidelines [1].

**Table 1:** Categories of videolaryngoscopes

**Type of videolaryngoscope**	**Description**	**Examples**
Macintosh-style videolaryngoscope (Macintosh-style VL)	– Macintosh-style bladesand video technology– Similar insertionprocedure to theMacintosh laryngoscope – A tube introducer andlaryngeal pressure areoften needed to clearlyvisualize the glottis	– Storz V-MAC – C-MAC – McGrath MAC
Hyperangulated videolaryngoscope (HA-VL)	– j-shaped orhyperangulated bladeable to visualize theglottis without flexing orextending the neck – Offers an indirect view ofthe glottis, and intubationrequires a pre-shapedstylet – Upper airway traumacould occur when the tipis not visible	– Glidescope – McGrath Series 5 – TruView Devices – Bullardlaryngoscope – Storz D-blade – AP Venner scope
Channeled-videolaryngoscope (Channeled-VL)	– j-shaped orhyperangulated bladewith a canal that guidesthe tracheal tube to theglottis – The tube tip is visiblethroughout trachealintubation	– Airtraq – Pentax-airwayscope

## Background of videolaryngoscopy in obstetrics

Given the peculiarities of the obstetric airway, the emergent nature of the vast majority of obstetric intubations, and the reality that difficult or failed intubation is mostly unpredictable in the parturient, there is a compelling need to reappraise current practices in obstetric airway management. Since Tunstall’s landmark guideline on failed obstetric intubation [26], several developments have been made to improve the safety of obstetric general anesthesia. One significant development in recent decades is the evolution of the videolaryngoscope (VL).

Initially, introduced in 2001 by Canadian surgeon Dr. John Pacey, the VL is arguably the most significant milestone in tracheal intubation since the invention of the Macintosh laryngoscope approximately 80 years ago. A VL is a laryngoscope equipped with a high-resolution digital camera located a few centimeters near the tip of a blade and a means of transmitting the image to a display, enhancing visualization of the larynx by providing a more anterior view of the glottis and a wider angle of vision [1, 27]. VLs are available in different sizes and blade shapes and may be portable, single-use or reusable, channeled or non-channeled [27]. Based on the shape of the blade and other operational mechanisms, three major categories of VLs have been recognized (Macintosh-style VL, hyperangulated [HA]-VL, and channeled VL), as summarized in Table 1 [1, 28].

When compared to a direct laryngoscope, the VL offers many benefits. The operator’s field of view during direct laryngoscopy is only 10–15 degrees, but videolaryngoscopy expands the angle of view to approximately 60–80 degrees [1], significantly improving the Cormack and Lehane (C/L) grades. Asai et al. [29] reported that in all but 1 of 256 patients with C/L grade 3 or 4 with the Macintosh laryngoscope, the view with the Pentax-AWS^®^-VL was either grade 1 or 2. Patients with cervical spine immobilization benefit greatly from the VL because its video camera eliminates the requirement for aligning the three airway axis and delivers a superior glottic view with less strain and cervical spine manipulation [27, 30, 31]. The shared airway view provided by VL facilitates teamwork, communication, and teaching during intubation and other aspects of airway care and substantially improves the margin of safety in airway management [27]. Furthermore, the study of Vuolato et al. [32], which investigated the differences in subjective and objective cognitive workload between HA-VL (Glidescope) versus direct laryngoscopy in a real clinical setting found that a videolaryngoscopy significantly reduced both measures; hence, proficiency with HA-VL constitutes an ergonomic option that could limit operators’ workload and improve patients’ safety. The Cochrane meta-analysis of Hansel et al. [33], which included more than 200 randomized controlled trials and more than 26,000 patients, concluded that VLs of any design reduce the rates of failed intubation and increase the rates of successful first-attempt intubation. Garcia et al. [34] studied first-attempt intubation success among emergency medicine trainees by laryngoscopic device and training year and found that each laryngoscopy device class was associated with improvement in first-attempt success as training progressed. The VL outperformed the direct laryngoscope for all operator groups, and postgraduate year-1 trainees achieved higher first-attempt success using a Macintosh style-VL than postgraduate year-3+ trainees using a direct laryngoscope. The authors supported the suggestion that a direct laryngoscope should not be routinely used for the first intubation attempt in adult patients unless clinical circumstances would favor its success [34]. Sakles et al. studied almost 2000 patients in their emergency department and reported a higher first-pass success intubation rate when using a GlideScope^®^-VL with an HA-blade in either a clean (91%) or soiled (81%) airway, compared with direct laryngoscope, with which first-pass success was 76% in a clean and 66% in a soiled airway. Therefore, whilst soiling of the airway reduced first-pass success by approximately 10% with both devices, the relative efficacy of videolaryngoscopy increased and was 21% more likely to achieve first-pass success in the clean airway and 24% more likely in the soiled airway [35].

Despite the aforementioned advantages of videolaryngoscopy, several limitations have been noted. In real-world airway management in obstetrics, a recent large observational study by Odor et al. [36] highlighted an alarmingly low adoption of videolaryngoscopy (1.9%). One important drawback is that a successful tracheal tube insertion is not always guaranteed, even when a C/L grade 1 view is demonstrated [37]. This phenomenon has been described as “laryngoscopy paradox” [38], predominantly associated with the use of HA-VL due to poor insertion technique in the vast majority of cases. Moreover, the presence of airway pathology, a history of airway surgery or neck radiotherapy, the presence of a cervical collar, and a restricted cervical range of motion are all variables that have been linked to failed videolaryngoscopy [30]. In addition, technical issues, like monitor malfunction, low battery power, solar glare, fogging, and the presence of fluids like blood or secretions in the airway, may blur or obscure the video screen image and, consequently, complicate intubation [27]. Perforation or laceration injuries to the palatopharyngeal wall, soft palate, and tonsillar pillar have been described with videolaryngoscopy [39–41], most likely associated with poor training/technique.

Several clinical recommendations have been proposed for improving techniques of videolaryngoscopy [42]. The HA-VL may be placed into the patient’s mouth either in the midline or along the right side of the tongue, watching the blade tip as it enters the mouth and continuing to monitor it on the VL screen after it disappears from view. Optimizing the laryngeal view by mentally dividing the VL screen into a 3 × 3 grid of 9 rectangles and locating the vocal cords in the center rectangle is crucial. Using a prefabricated stylet, a curved malleable stylet, or a stylet that can be “activated” into a curved shape, the tracheal tube should be fashioned into a shape that roughly resembles the curvature of the HA-VL. A tracheal tube half a size smaller than normal should be used, and a reinforced straight tracheal tube may be appropriate. Lubricant should be applied to the stylet and the end of the tracheal tube. When the tip of the tracheal tube is moved along the hyperangulated blade, it will consistently and effortlessly pass to the location the camera is pointed (i.e., into the glottis). This eliminates the potential for injury of the pharyngeal wall caused by a blind spot. As the stylet tube is advanced to the glottis, the stylet should be retracted. When advancing the tracheal tube, if there is any resistance, it can be turned 30–45 degrees clockwise or counterclockwise. Intubation with a bougie is not advised since the device unfurls too quickly, increasing the likelihood of a botched procedure due to insufficient curvature [42]. A simplified airway management algorithm has been introduced to facilitate the clinical adaptation of VL and the teaching of protocol to novices [43]. In the absence of a predicted difficult airway which dictates the use of HA-VL, the Macintosh-style VL is suggested as a primary intubation device as it offers the benefits of videolaryngoscopy and direct laryngoscopy [44]. Meanwhile, considering the great diversity of VL models, it may be prudent that each institution selects one or a maximum of two models of VLs to limit the challenges associated with the multiplicity of devices.

### Videolaryngoscopy vs direct laryngoscopy in obstetrics: Comparison of primary outcomes

Much of the current evidence on the use of videolaryngoscopy in obstetrics derives from the considerable experience and the broad spectrum of studies in non-obstetric airway management [45]. An extensive literature search was performed on Medline (PubMed) using different combinations of keywords and vocabulary terms related to the subject of the review (including *obstetrics*, *pregnancy*, *videolaryngoscopy*, *intubation*, and *general anesthesia*). The following search criteria were applied: full-text-accessible articles, articles in English, peer-reviewed original research reports or relevant systematic reviews, without restriction to the year of publication. Additional literature was sourced by examining the reference lists of all studies extracted from the database search. Altogether, the literature search yielded a limited body of studies that specifically examined the use of videolaryngoscopy in the obstetric population, with a preponderance of case reports/series and observational studies. A few randomized control trials evaluated some clinical outcomes in the application of videolaryngoscopy in obstetrics [46–48]. Superior glottic visualization higher (percentage of glottic opening [POGO]) value and favorable C/L grade was demonstrated with different VL models compared to the Macintosh laryngoscope in orotracheal intubation [46, 48]. However, a recent meta-analysis of three randomized-controlled trials involving obstetric patients without difficult airways demonstrated no disparity between videolaryngoscopy and direct laryngoscopy regarding first-attempt success rate and time to tracheal intubation [49].

In a retrospective analysis of 180 obstetric intubations, Aziz et al. reported that first-pass intubation was successful in 157 of 163 patients (95% CI 92%–99%) using a direct laryngoscope, while first-attempt intubation was successful in 18 of 18 cases (95% CI 81%–100%) with videolaryngoscopy, with most patients managed by videolaryngoscopy exhibiting predictive features of a difficult airway. However, the findings of this study should be interpreted cautiously, given that the incidence of failed intubation was not large enough to conclude whether videolaryngoscopy improves outcomes [50]. Similarly, an observational study of 100 intubations in an obstetric unit reported that videolaryngoscopy was the modality of choice in two-thirds of cases, and successful intubation was recorded in all cases [51]. In addition, several case reports have also described the successful use of videolaryngoscopy in the setting of difficult airways or as a rescue modality following failed direct laryngoscopy in parturients with challenging airways [52–55].

In patients undergoing elective cesarean section under general anesthesia, it was found that hemodynamic parameters were better preserved in the first three minutes with the GlideScope^®^-VL compared to the Macintosh direct laryngoscope, although other outcome measures (Mallampati score, sore throat, Apgar scores, and hemodynamic changes after three minutes) were similar in both groups [47]. It has similarly been suggested that videolaryngoscopy may be associated with more attenuated sympathetic response in hypertensive obstetric patients [56].

A randomized comparison of two VL models (C-MAC and King Vision^®^) for obstetric intubation noted that while both models did not differ from direct laryngoscopes regarding time to intubation, the C-MAC-VL may be preferable for obstetric intubations due to favorable performance indices such as ease of use and less need for optimization maneuvers [48].

## Current practice guidelines recommendations on the role of videolaryngoscopy in obstetrics

Until recently, there has been a consistent failure to recognize the peculiarities of the obstetric airway in most of the major airway management guidelines. For example, in the Task Force guidelines for the management of the difficult airway by the Difficult Airway Society UK (DAS) and American Society of Anaesthesiologists (ASA), no specific consideration was accorded to the obstetric patient [2]. The 2022 ASA Practice Guideline for Management of the Difficult Airway [57] extensively reviewed the published randomized-controlled trials on the use of the videolaryngoscopy in airway management, and reported Category A1-B evidence that videolaryngoscopy was superior to direct laryngoscopy with regard to laryngeal views, the success rate for intubation, rate of first attempt intubation, and the number of intubation manoeuvres; with equivocal findings for time to intubation (Category A1-E evidence). The obstetric population was not explicitly considered in the analysis and report of the current evidence.

However, in recent years, tailored guidelines for airway management in obstetrics have emerged. The Obstetric Anaesthetists’ Association and Difficult Airway Society Guideline for the Management of Difficult and Failed Tracheal Intubation in Obstetrics [2] is perhaps the most widely referenced of the tailored obstetric guidelines. The Guidelines recommend that “a VL should be immediately available for all obstetric general anesthetics.” Nevertheless, it also points out several current limitations, including the lack of reliable comparative studies on the most suitable VL model for obstetric intubation, the phenomenon of “laryngoscopy paradox,” and reports of traumatic laryngoscopy, especially with stylet devices.

The All-India Difficult Airway Association 2016 Guidelines for the Management of Unanticipated Difficult Tracheal Intubation in Obstetrics [58] recommends that in accordance with local practice and expertise, laryngoscopy may be performed with either a direct laryngoscope or a VL. The Guidelines, however, recommend that in the event of an unsuccessful first attempt, “the second attempt at laryngoscopy should be performed using a VL, alternate blades and use of a bougie as dictated by the availability of equipment and expertise.” Using a VL in such a situation allows the assistant to gradually decrease cricoid pressure while monitoring the effect on the laryngoscope’s field of view [58]. However, if tracheal intubation on the first attempt using a direct laryngoscope is difficult, one will have “wasted” an attempt that might have been more easily salvaged with a Macintosh-like VL +/– adjunct by just looking at the screen after using it as a direct laryngoscope. In a comparative analysis of commonly used rescue intubation techniques after failed direct laryngoscopy, Aziz et al. [59] reported a high rescue intubation success rate with videolaryngoscopy. However, the retrospective observational nature of this study should be considered in interpreting the frequency of airway rescue and the rescue success rates reported [59].

## Challenges of clinical adoption of videolarygoscopy in obstetrics

Besides the already highlighted limitations, such as inconsistencies in comparative clinical outcomes, a few other issues warrant further consideration regarding the routine use of VLs in obstetrics. There is little or no evidence regarding the predictors of difficult videolaryngoscopy in obstetrics, and there is no clear protocol for managing failed videolaryngoscopy [27]. The exact factors that facilitate successful tracheal tube insertion with videolaryngoscopy have been insufficiently characterized; hence, improved glottic visualization does not always translate to successful intubation. The multiplicity of VL models with unique operational protocols poses a challenge to learning and uniformity of practice, and the evidence remains limited to guide the rational choice of the appropriate type of device according to indication or context [60]. Additionally, the benchmark for expertise and the appropriate pedagogical approach for videolaryngoscopy remains uncertain.

Reports regarding the learning curve for VLs are conflicting. For example, in a prospective trial with 40 intubation-naive medical students in simulated simple and difficult laryngoscopy scenarios, Maharaj et al. [61] reported that the Airtraq-VL was associated with a rapid learning curve compared to the Macintosh laryngoscope and, accordingly, appears more suitable for teaching tracheal intubation to novices. In contrast, Cortellazzi et al. [62] reported that expertise with the VL was attained after 76 attempts, suggesting that prolonged experience and training were necessary to attain proficiency with the device. Basic intubation competencies appear to be retained over time independent of the laryngoscopic method; however, videolaryngoscopy in the hands of novice operators was associated with quicker intubation and fewer adverse events after three months with no additional intubation training. Additionally, it was shown that while first-attempt success with direct laryngoscope did not increase significantly over the course of residency training, the performance of videolaryngoscopy improved substantially [64].

Furthermore, given the proliferation of VL models, each with unique technical properties and operational requirements (e.g., patient positioning, tongue displacement, insertion depth, the direction of applied forces, blade axis, etc.), successful intubation may only be practical with technical competence with each specific device, and familiarity with the use of different devices may require a long learning curve [1]. Simply put, expertise with the traditional Macintosh laryngoscope is not transferable to the VL, and mastery of one VL model does not translate to proficiency with other models [65]. This particularly applies to the technique required the use of an HA-VL blade, which is completely different from that of a Macintosh-shaped laryngoscope blade. When an intubator gains a good view of the larynx using a hyperangulated blade but fails to use an appropriate technique, it is likely that the intubator and not the device is the cause of the unsuccessful intubation [42].

Finally, the enormous cost disparity between VLs and conventional laryngoscopes remarkably restricts the availability and use of the former, especially in the resource-limited settings [27]. Although, in recent years, notably since the beginning of the COVID-19 pandemic, there has been an increasing availability of low-cost generic and handcrafted VL models [66, 67]. There are concerns regarding quality and evidence for their comparability with validated models is limited. On the other hand, a broader consideration of the health economics of videolaryngoscopy use in surgical settings has shown that, in reality, it may be associated with a reduction in the total cost of inpatient care, the length of hospital stay, and the odds of procedural complications and intensive care unit admission [68]. Accordingly, equipment and training costs may be substantially buffered by overall reductions in healthcare costs that may be associated with the adoption of videolaryngoscopy. Moreover, the COVID-19 pandemic has increased awareness of the risk involved in managing the airway with conventional laryngoscopy, which may contribute to overcoming previous reluctance to the routine use of videolaryngoscopy [69].

## Conclusion

Videolaryngoscopy enhances glottic visualization, increases the odds of successful intubation, and promotes team communication and teaching during intubation. In cases of unexpected or difficult airway, or unsuccessful direct laryngoscopy, videolaryngoscopy has proven effective [28]. Considering the anatomical and physiological peculiarities of the obstetric airway, the poor predictive value of routine airway clinical assessment tests, and the fact that most obstetric intubations occur in emergency settings, it may be prudent to assume that most obstetric intubations will be difficult, and VL provides compelling advantages in this regard. The Macintosh-style VL can be suggested as a primary intubation device as it offers the benefits of videolaryngoscopy and direct laryngoscopy. However, more rigorous evidence is urgently needed to clarify the current blind spots and controversies regarding the use of videolaryngoscopy in obstetrics and other clinical settings.
